# Motion Control of a Gecko-like Robot Based on a Central Pattern Generator

**DOI:** 10.3390/s21186045

**Published:** 2021-09-09

**Authors:** Qing Han, Feixiang Cao, Peng Yi, Tiancheng Li

**Affiliations:** 1 School of Robot Engineering, Yangtze Normal University, Chongqing 408100, China; hanqing@mail.nwpu.edu.cn (Q.H.); 20130030@yznu.edu.cn (P.Y.); 2 School of Mechatronic, Northwestern Polytechnical University, Xi’an 710072, China; caofeixiang@mail.nwpu.edu.cn; 3 School of Automation, Northwestern Polytechnical University, Xi’an 710072, China; 4 Air Institute, IoT Digital Innovation Hub, 47011 Valladolid, Spain

**Keywords:** gecko-like robot, central pattern generator, motion control, collision avoidance

## Abstract

To solve the problem of the motion control of gecko-like robots in complex environments, a central pattern generator (CPG) network model of motion control was designed. The CPG oscillation model was first constructed using a sinusoidal function, resulting in stable rhythm control signals for each joint of the gecko-like robot. Subsequently, the gecko-like robot successfully walked, crossed obstacles and climbed steps in the vertical plane, based on stable rhythm control signals. Both simulations and experiments validating the feasibility of the proposed CPG motion control model are presented.

## 1. Introduction

Geckos are known for their excellent ability to overcome obstacles, climb walls and run on ceilings [1]. Gecko-like robots, which can move and work on a vertical wall, are popular and used worldwide [2,3], and can be employed for various tasks, such as antiterrorism activities, post-disaster rescue, engineering tests, and maintenance and inspection in hazardous environments [4,5,6,7]; however, the locomotion control of gecko-like robots is a challenging project, and the performance of gecko-like robots is still not good enough to meet most requirements. This has attracted immense attention from academics and corporate executives [8,9,10,11] around the world.

The motion control methods of a legged robot are usually based on its model, its behavior, or the methods of its central pattern-generator [12,13,14,15,16]. The model-based method is able to achieve high-precision motion in a known environment but leads to poor adaptability in unknown environments. The behavior-based method offers good adaptability in dynamic unknown environments but makes handling the complex task of global planning difficult. The CPG-based method avoids the tedious modeling process of the model-based control method. It can coordinate multiple degrees of freedom to generate multimodal motion by adjusting a few parameters, and it offers good self-stability and adaptability. Coordinated multi-degree-of-freedom (DOF) motion control based on CPG has many advantages and has become an important topic in the field of bionic robot control, even though multi-DOF robots are slow and consume large amounts of energy. It is crucial to address the gait design of multi-DOF robots. The rhythmic movements of animals, such as walking, running, swimming and flying, are implemented by the central pattern generator [17]. Venkataraman, S.T. [18] developed a simple six-legged-robot locomotion gait model by using CPG; Zhang, Z.G. et al. [19] designed a four-legged walking robot using CPG in an unstructured environment; Kimura, H. et al. [20] achieved gait control for a quadruped robot using CPG neuron flexor and extensor muscles; and Inagaki, S. et al. [21] built a mathematical model of CPG and exploited a hardware circuit to realize multiple robot control using CPG. Moreover, Ijspeert A.J. et al. [22,23] achieved newt-like robot control based on the CPG method. By changing the oscillator frequency of each joint of the robot’s trunk changed the body track from the standing wave of the crawling gait to the traveling wave of the swimming mode, they designed two newt-like modes of motion, i.e., crawling on land and swimming in water. Antos et al. [24] established a CPG model using layered thought to achieve quadruped robot control. Changing the coupling parameters of the phase difference and duty cycle transformed the motion of the quadruped robot from a crawling gait to a diagonal gait, and it was possible to switch smoothly between the two. Fan, J. Z. et al. [25] used a variable topology CPG network model with a feedback mechanism to make a snake robot roll, crawl, and cross different obstacles. Donati, E. et al. [26] described the preliminary results of a spiking implementation of the lamprey Central Pattern Generator (CPG) using Neuromorphic VLSI devices. Arena P. et al. [27] studied an autonomous, biologically inspired swimming robot based on the paradigm of the Central Pattern Generator, which was capable of mimicking the undulatory swimming of a sea lamprey. Stefanini, C. et al. [28] tested the CPG hypotheses and investigated goal-directed locomotion by exploiting visual input from a vision system that processed video streams. Ijspeert A.J. et al. [29] studied a control architecture for controlling the locomotion of an amphibious snake/lamprey robot capable of swimming and serpentine locomotion using a lamprey-like central pattern generator mode.

Gecko-like robots have the distinct advantage of strong adaptability in unknown dynamic environments. Therefore, for this study we designed the structure of a gecko-like robot and planned its corresponding movement gait to produce movement in a complex environment. The gecko-like robot in this study used 12 CPG oscillators to control four leg-lifting joints, telescopic leg joints and electromagnetic suction cups. In order to coordinate this multi-joint motion, it was necessary to construct a phase relationship between 12 oscillators and form an oscillation network. If 12 CPG oscillators were connected in parallel at the same time, this would increase the complexity of the CPG network model and reduce the expansion performance and universality of the network model. In this study, the phase relationship between 12 CPG oscillators was divided into three stages. The first-stage phase relationship consisted of four CPG oscillators with leg-lifting joints; the second-stage phase relationship consisted of four CPG oscillators with telescopic leg joints; and the third-stage phase relationship consisted of four CPG oscillators with electromagnetic suction cups.

In order to achieve good simulation results, some constraints, including fixed constraints, rotation constraints, ball hinge pair and spring constraints, adsorption force constraints and contact force constraints, needed to be imposed. Under these constraints, the gaits adopted by the gecko-like robot, such as walking, turning motion, obstacle-crossing, and step-climbing, were planned. The experimental system included steel plate 1, steel plate 2, an obstacle and the robot. Steel plate 1 and steel plate 2 were made of ordinary carbon steel with a width of 400 mm, and the inclination (θ) between the steel plate and horizontal plane could be adjusted. Steel plate 2 and steel plate 1 were connected by bolts to form a step with a height of 8 mm. The size of the obstacle was 12 mm × 12 mm × 220 mm. The adsorption method was electromagnet adsorption, and the maximum adsorption force of the electromagnet was 25 N. In simulations and experiments, the gecko-like robot successfully achieved walking, obstacle-crossing, and step-climbing in the vertical plane, based on the stable rhythm control signals. The simulations and experiments validated the feasibility of the CPG motion control model.

In sum, this paper provides the following contributions:

(a) A central pattern generator (CPG) network model of motion control was designed, and the CPG oscillation model was constructed using a sinusoidal function, so that the stable rhythm control signals of each joint of the gecko-like robot could be generated.
(b) The gecko-like robot in this study used 12 CPG oscillators to control four leg-lifting joints, telescopic leg joints and electromagnetic suction cups. By connecting 12 CPG oscillators in parallel at the same time, it was possible to increase the complexity of the CPG network model and reduce the expansion performance and universality of the network model. In this paper, the phase relationship between the 12 CPG oscillators was divided into three stages. The first-stage phase relationship consisted of four CPG oscillators with leg-lifting joints; the second-stage phase relationship consisted of four CPG oscillators with telescopic leg joints; and the third-stage phase relationship consisted of four CPG oscillators with electromagnetic suction cups.
(c) Control of the gecko-like robot was based on a sinusoidal oscillation model. The oscillator produced stable and smooth sinusoidal control signals without external feedback. When the oscillator received external feedback or a high-level decision-making system regulation signal, it quickly adjusted the oscillation period, phase and amplitude, and the biological characteristics of the CPG were simulated very well.
(d) A curve interpolation method was proposed to fit the angle time curve, in order to achieve accurate control of the multi-channel steering gear without delay.
(e) The gecko-like robot successfully managed walking, obstacle-crossing, and step-climbing in the vertical plane, based on the stable rhythm control signals, and our simulations and experiments validated the feasibility of the CPG motion control model. More importantly, the method proposed in this paper can be applied to other bionic robots, such as multi-legged robots and the lamprey robot.

The remainder of the paper is organized as follows. Section 2 describes the structure of the gecko-like robot. Section 3 presents the motion control of the gecko-like robot based on CPG. The simulation and experiment results are given in Section 4. In Section 5, we offer our conclusions.

## 2. Materials and Methods

**Figure 1:**
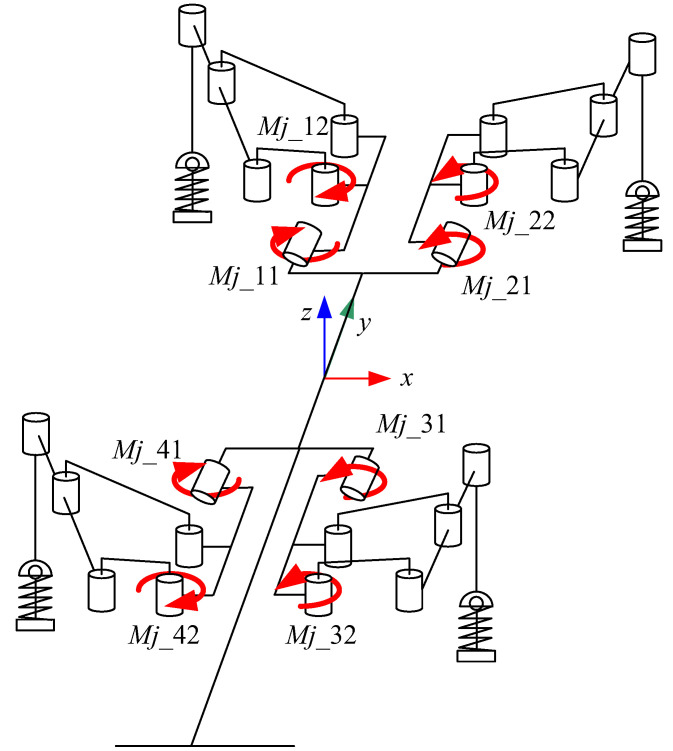
Mechanism of the gecko-like robot.

The gecko-like robot was made of plexiglass with a symmetrical four-legged distribution and a simple structure. Each leg had two active joints and one driven joint. The active joint, driven by a steering engine, was a four-bar parallel structure, which improved the rigidity and reliability of the robot. Adding an elastic damping link to the driven joint helped to adjust the landing position of the robot over a small range so that the robot had good ground adaptability. The robot had 10 driving joints, which drove the parallel four-bar mechanism to achieve leg-lifting, leg-stretching, waist-bending and tail-stretching. The mechanism of the gecko-like robot is shown in Figure 1, and the main parameters of the gecko-like robot are shown in Table 1.

## 3. Motion Control Based on CPG

First, the CPG control method automatically generated stable rhythm signals in the absence of high-level regulation and external feedback, but the high-level decision-making, or feedback signals, also adjusted the output signals of the CPG, thus increasing the adaptability of the gecko-like robot to known or unknown environments. Second, the CPG generated a variety of control signals with different phase differences through phase self-locking or interlocking; thus, multi-degree-of-freedom coordinated motion was achieved under different motion modes and behavior conditions. Finally, the structure of the CPG was simple and scalable, which facilitated the evolution of the complex motion behavior of the gecko. Therefore, the use of CPG as the bottom motion controller of gecko-like robots is of great significance; this is especially true when it is combined with the method proposed in recent research [30].

Typical gaits adopted by quadruped robots include walking, jogging, walking and running. When a gecko-like robot walks on a wall, the robot must keep more than two legs in contact with the wall at the same time. Given the symmetrical structure of the gecko-like robot, the trotting gait (diagonal) was chosen as the basic walking gait of the robot. When the robot walked, the sensor detected discontinuous terrain such as obstacles or steps on the wall. The robot’s senior decision-making level made a judgment and triggered the gecko-like robot to switch to obstacle-crossing mode or step-crossing mode.

### 3.1. CPG Model

**Figure 2:**
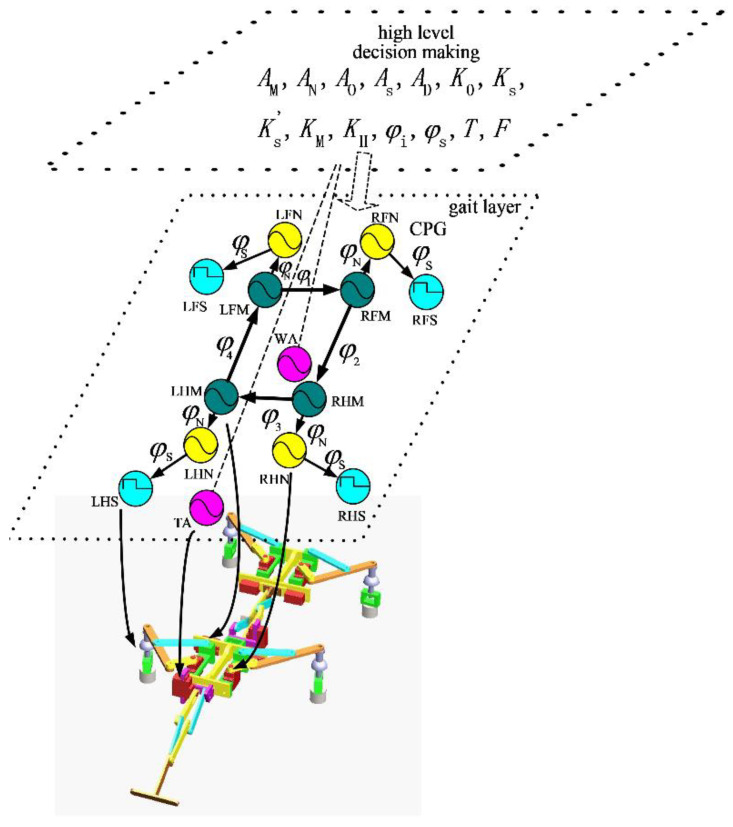
Overall diagram of the CPG control system.

At present, a commonly used CPG oscillator model is the Matsuoka model discussed by Lewis, et al. [17]. The CPG oscillator is established by simulating neurons, which are composed of two neurons, corresponding to the control of flexor and extensor muscles of animals. The outputs of the two neurons inhibit each other, and an external constant input and feedback input are introduced. Therefore, the oscillator has some shortcomings, such as its complex structure, the difficulty of setting its parameters and the complexity of its dynamic characteristic analysis, and its expression is not intuitive in the macro-physical sense. In our study, control of the gecko-like robot was based on a sinusoidal oscillation model. The oscillator produced stable and smooth sinusoidal control signals without external feedback. The CPG network was constituted by the interlock phase, which is shown in Figure 2 (meanings of signs: L—left, R—right; F—front, H—back, M—leg lifting, N—telescopic legs, S—osculum, W—waist, and T—tail). Four oscillators acted on the leg-lifting joint, four oscillators acted on the leg-expanding joint, four oscillators were converted into square wave signals to control the sucker, and two oscillators acted on the waist and tail of the robot. The variables on the joint line of the oscillators represent the phase difference, and the arrow pointing to the joints indicates lagging in the phase.

When the wall-climbing robot walked on a non-feedback plane, the swing trajectory of the leg-lifting joint was half a sinusoidal period. The CPG oscillation model in half-wave form is shown as Equation (1):

(1) θ(t)=max(Asin(2πt/T+φ),0)

For the robot controlled by electromagnetic chuck switching value, the designed adsorption CPG oscillation model is shown as Equation (2):

(2) F(t)=F(AINT(t/T)modT)

The mathematical expression of the CPG network model for the gecko-like robot with 12 CPG oscillators is depicted in Equation (3). The values of each parameter are given in Table 2.

(3) $$\{\begin{array}{c} \theta_{Mi}=\{\begin{array}{c} A_{D}A_{M}\sin(\frac{2\pi t}{T}+\varphi_{i})+K_{0}A_{0}\sin(\frac{2\pi (t-t_{0})}{T_{0}})+K_{S}(A_{S}+K_{S}^{'}A_{S}\sin(\frac{2\pi (t-t_{S})}{T}))K_{M}=0\\ \max(A_{M}\sin(\frac{2\pi t}{T}+\varphi_{i})+K_{0}A_{0}\sin(\frac{2\pi (t-t_{0})}{T_{0}})+K_{S}(A_{S}+K_{S}^{'}A_{S}\sin(\frac{2\pi (t-t_{S})}{T})),0)\;K_{M}=1 \end{array}\\ \theta_{Ni}=A_{N}\sin(\frac{2\pi t}{T}+\varphi_{i}+\varphi_{N})\\ F_{i}=\{\begin{array}{c} F(\mathrm{AINT}((t+\frac{\varphi_{S}}{T}\pm \frac{\Delta t}{2})\mathrm{mod}T))\;\;\;\;\;\;\;\;\;K_{H}=0\\ 0\;\;\;\;\;\;\;\;\;\;\;\;\;\;K_{H}=1 \end{array} \end{array}$$

When the robot walks with a diagonal gait, $K_{0}=0$, $K_{S}=0$ and A_D_ = 1, and Equation (3) can be simplified as (4):

(4) $$\begin{array}{l} \left\{\begin{array}{c} \theta_{Mi}=\{\begin{array}{c} A_{D}A_{M}\sin(\frac{2\pi t}{T}+\varphi_{i})+K_{0}A_{0}\sin(\frac{2\pi (t-t_{0})}{T_{0}})+K_{S}(A_{S}+K_{S}^{'}A_{S}\sin(\frac{2\pi (t-t_{S})}{T}))\;\;\;K_{M}=0\\ \max(A_{M}\sin(\frac{2\pi t}{T}+\varphi_{i})+K_{0}A_{0}\sin(\frac{2\pi (t-t_{0})}{T_{0}})+K_{S}(A_{S}+K_{S}^{'}A_{S}\sin(\frac{2\pi (t-t_{S})}{T})),0)\;K_{M}=1 \end{array}\\ \theta_{Ni}=A_{N}\sin(\frac{2\pi t}{T}+\varphi_{i}+\varphi_{N})\\ F_{i}=\{\begin{array}{c} F(\mathrm{AINT}((t+\frac{\varphi_{S}}{T}\pm \frac{\Delta t}{2})\mathrm{mod}T))\;\;\;\;\;\;\;\;\;K_{H}=0\\ 0\;\;\;\;\;\;\;\;\;\;\;\;\;\;K_{H}=1 \end{array} \end{array}\right.\\ =\{\begin{array}{c} \theta_{Mi}=\{\begin{array}{c} 1*A_{M}\sin(\frac{2\pi t}{T}+\varphi_{i})+0+0\;\;\;\;\;\;\;\;\;K_{M}=0\\ \max(A_{M}\sin(\frac{2\pi t}{T}+\varphi_{i})+0+0\;\;\;\;\;\;\;\;K_{M}=1 \end{array}\\ \theta_{Ni}=A_{N}\sin(\frac{2\pi t}{T}+\varphi_{i}+\varphi_{N})\\ F_{i}=\{\begin{array}{c} F(\mathrm{AINT}((t+\frac{\varphi_{S}}{T}\pm \frac{\Delta t}{2})\mathrm{mod}T))\;\;\;\;\;\;\;\;K_{H}=0\\ 0\;\;\;\;\;\;\;\;\;\;\;\;\;K_{H}=1 \end{array} \end{array}\\ =\{\begin{array}{c} \theta_{Mi}=\{\begin{array}{c} A_{M}\sin(\frac{2\pi t}{T}+\varphi_{i})\;\;\;\;\;\;\;\;\;\;K_{M}=0\\ \max(A_{M}\sin(\frac{2\pi t}{T}+\varphi_{i})\;\;\;\;\;\;\;\;\;K_{M}=1 \end{array}\\ \theta_{Ni}=A_{N}\sin(\frac{2\pi t}{T}+\varphi_{i}+\varphi_{N})\\ F_{i}=\{\begin{array}{c} F(\mathrm{AINT}((t+\frac{\varphi_{S}}{T}\pm \frac{\Delta t}{2})\mathrm{mod}T))\;\;\;\;\;\;\;\;K_{H}=0\\ 0\;\;\;\;\;\;\;\;\;\;\;\;\;K_{H}=1 \end{array} \end{array} \end{array}$$

When the robot moves with a turning gait, $K_{0}=0$ and $K_{S}=0$, and, using the above method, Equation (3) can be simplified as (5):

(5) $$\{\begin{array}{c} \theta_{Mi}=\{\begin{array}{c} A_{M}\sin(2\pi t/T+\phi_{i}\;)\;\;\;\;\;\;\;\;\;\;\;\;\;\;\;\;\;\;\;\;\;K_{M}=0\\ \max(A_{M}\sin(2\pi t/T+\phi_{i}\;)\;,0)\;\;\;\;\;\;\;\;\;\;\;\;\;\;\;\;\;\;\;K_{M}=1 \end{array}\;\\ \theta_{Ni}=A_{D}A_{N}\sin(2\pi t/T+\phi_{i}+\phi_{N})\\ F_{i}=\{\begin{array}{c} F(\mathrm{AINT}((t+\phi_{S}/T\pm \Delta t)\;\mathrm{mod}\;T))\;\;\;\;\;\;\;\;\;\;\;\;\;\;\;\;K_{H}=0\\ 0\;\;\;\;\;\;\;\;\;\;\;\;\;\;\;\;\;\;\;\;\;\;\;\;\;\;\;\;\;K_{H}=1 \end{array}\; \end{array}$$

### 3.2. Gait Planning

Gait planning is the planning of the trajectory of each joint according to the kinematic and dynamic characteristics of the wall-climbing robot. This is very important for the climbing robot, and two conditions are required. One is that the planned gait should conform to the walking habits of quadrupeds, and the other is that the planned gait should keep the robot walking stably on the wall.

The robot studied here was a gecko-like robot with movement based on the gait of quadrupeds. The gait quadrupeds refers to the phase relationship of four legs in the process of walking. It is mainly composed of a walking gait, a trotting gait, a galloping gait and a bounding gait [31]. Each gait is optimal in some aspect, such as speed, stability or energy consumption. The walking gait is a type of tripod supported gait, which offers the best stability, but the speed is slow and energy consumption is high. The initial walking gait of a quadruped animal is characterized by alternating, four-legged strides, and it involves at least three legs landing on the ground. The trotting gait is a two-phase gait with moderate speed and stability and low energy consumption, and it is the most common gait adopted by quadruped animals. In the trotting gait, the left foreleg and right hind leg move in the same phase, as do the right foreleg and left hind leg. The latter is known as the diagonal gait. In the pacing gait, the two legs on the same side move in the same phase and opposite legs move in separate phases. The pacing gait is usually used when the animal is anxious or tired. In the running gait, the two front legs and the two hind legs move in the same phase as each other, and legs on the same side as and diagonal to each other move in separate phases. This gait creates fast movement, but energy consumption is high. It is commonly adopted by animals running at a high speed. The small jump gait involves four legs moving in the same phase, leaving the ground and landing at the same time. The small jump gait generally occurs when animals are very excited, panicked or in other abnormal states. Since the various gaits offer different characteristics for use in different environments, robots need to switch gaits according to the situation in order to adapt to different environments and requirements [32].

### 3.3. Simulation Environment

In order to achieve good simulation results, some constraints, including fixed constraints, rotation constraints, ball hinge pair and spring constraints, adsorption force constraints and contact force constraints, needed to be imposed. In order to analyze the characteristics of the robot crawling on the wall, the trunk and servo actuator of the wall-climbing robot, as well as the components that needed to be connected by a fixed pair, needed to be connected by the fixed pair. The wall, wall obstacles and wall steps were established and were attached, using fixed constraints, to the geodetic coordinate system. In order to achieve driving speed, the rotation pair in each rotation joint and the CPG control function in the driven joint of the servo actuator were added. The ball joint pair and spring constraints connected the robot’s feet to the electromagnetic sucker, so that the robot had a good ability to adapt to the environment. The adsorption force was added to the electromagnetic sucker, and the CPG function was used to control the adsorption force. The contact force was a special kind of force acting on the component. The contact force was produced when two components made contact with each other and deformed. The contact force size was related to the size of the deformation and the sliding speed. The crawling robot walking on the ground was simulated based on the friction between the foot and the ground. Therefore, it was necessary to define how the contact between the electromagnetic sucker and the supporting surface, as well as the friction force, would be generated. The solid-to-solid contact form was selected, and the contact force between the four feet and the ground was defined in the simulation. The relevant parameters and descriptions are shown in Table 3.

### 3.4. Rectilinear Motion

**Figure 3:**
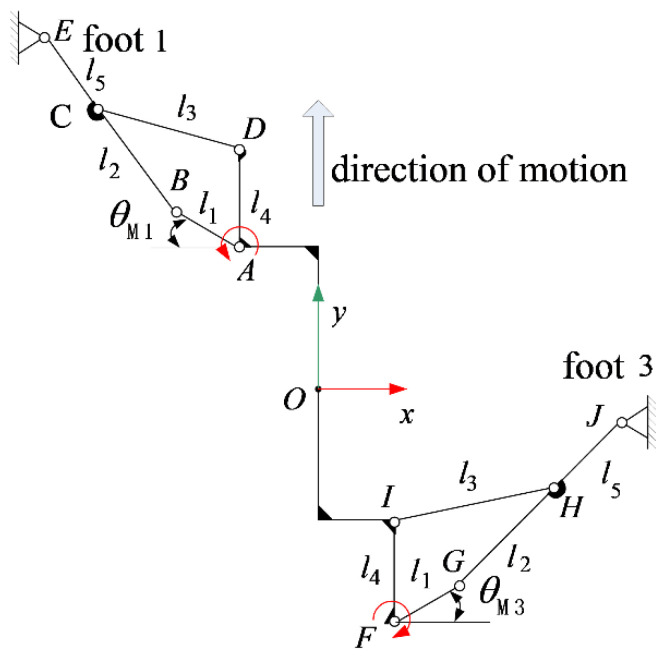
Analysis of the mechanism when the robot walked straight forward.

Regardless of the gecko-like robot’s feet skidding, the robot completed linear motion in a diagonal gait, requiring the diagonal support foot to swing in the same direction while reducing the swing in other directions. If foot 1 and foot 3 were in the support state, then foot 2 and foot 4 would be in the suspended swing state, and the leg-lifting joint would be in the static state. The diagonal gait linear motion analysis sketch of the gecko-like robot is shown in Figure 3.

When the robot walks in a diagonal gait, $K_{0}=0$, $K_{S}=0$ and $A_{D}=1$, Equation (3), can be simplified as (4), adjusting T and $A_{M}$ independently in Equation (4). The oscillation period and amplitude of the central nervous system is changed rapidly and steadily, and the walking speed and leg-lifting height of the robot could be adjusted. From the various movements, such as rectilinear motion, turning motion, striding over obstacles and climbing step, the robot’s straight walking gait was taken as an example. The basic motion process was as follows:

1. In the initial state, the four feet of the robot were in the adsorption state with the wall;
2. Robot feet 1 and 3 were adsorbed on the wall, while feet 2 and 4 were in the swing state;
3. Robot feet 2 and 4 lifted, and then swung forward. At the same time, legs 1 and 3, in the supporting state, swung backward and drove the body of the robot to move forward;
4. Feet 2 and 4 of the robot dropped, and were then adsorbed on the wall. At this time, the four feet were in the state of adsorption support;
5. Feet 2 and 4 of the robot were adsorbed on the wall, and feet 1 and 3 were in the swing state;
6. Feet 1 and 3 of the robot lifted, and then swung forward. At the same time, legs 2 and 4 in the supporting state swung backward, and then drove the body of the robot to move forward;
7. Feet 1 and 3 of robot dropped, and then feet 2 and 4 adsorbed with the wall. At this time, all four robot feet were in the adsorption support state.

**Figure 4:**
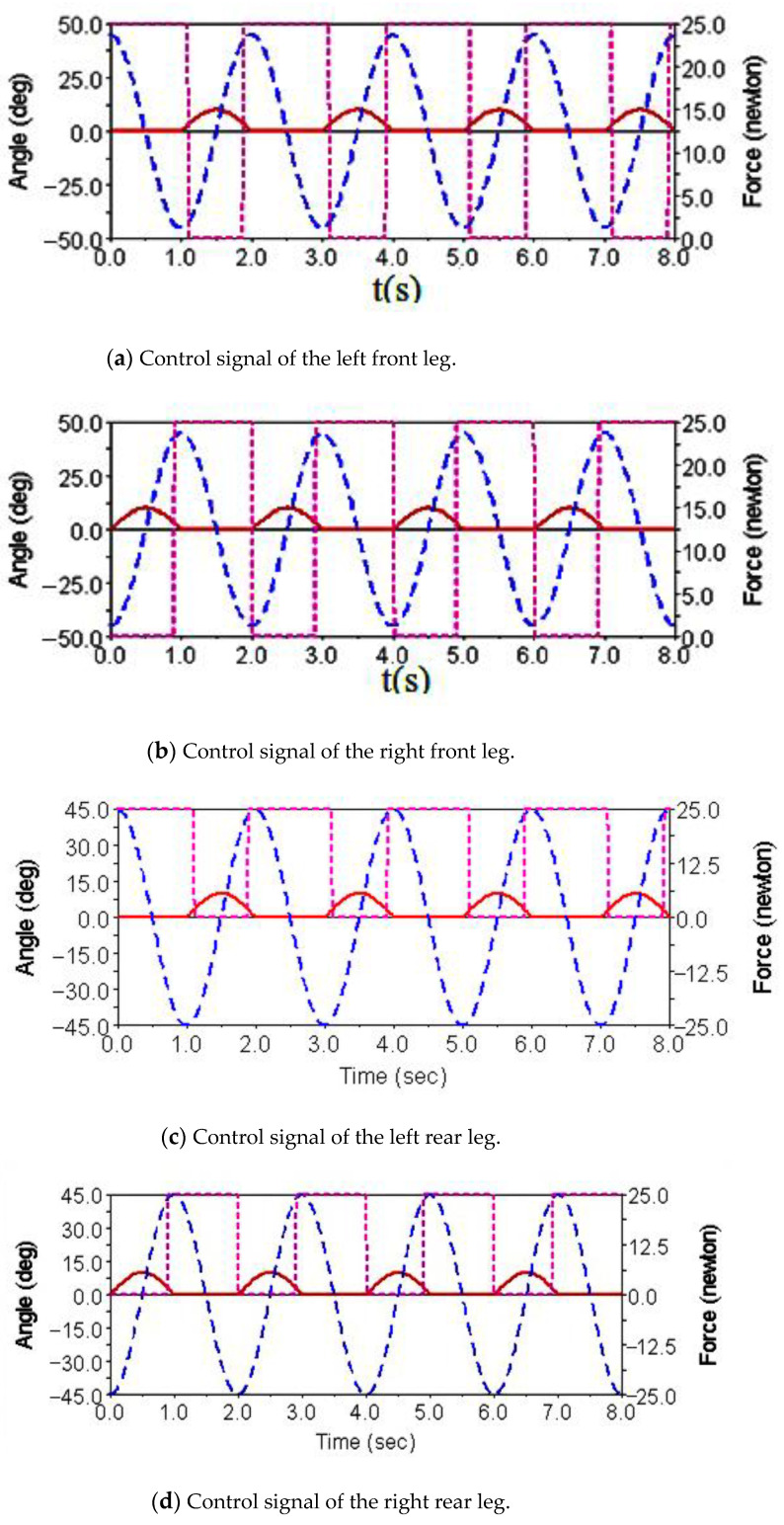
Robot leg control signals for diagonal gait and walking straight forward.

**Figure 5:**
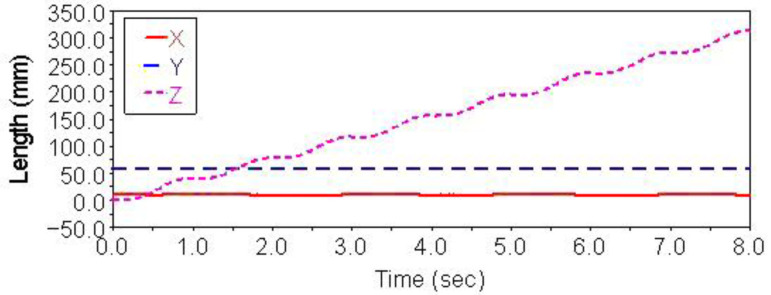
Centroid displacement curve of straight gait.

**Figure 6:**
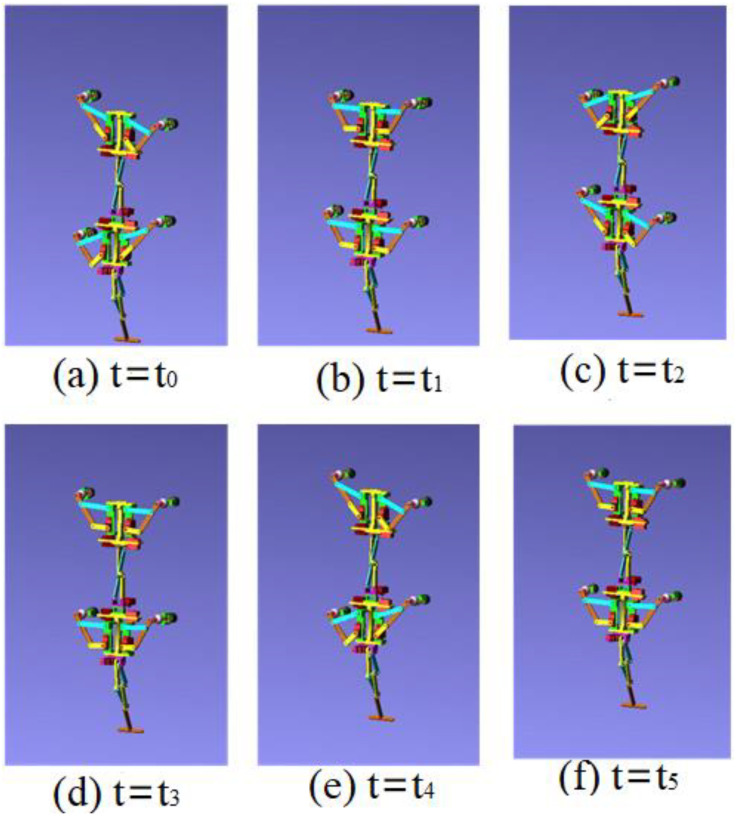
Diagonal gait for walking straight forward in ADAMS in different moments.

Gait simulation was carried out in the automatic dynamic analysis of the mechanical systems (ADAMS) environment. The control signals of each foot are shown in Figure 4. It can be seen from this Figure that the left forefoot was moved in the same phase as the right hind foot, and the right front foot moved in the same phase as the left hind foot. The ipsilateral forefoot and hindfoot phases differed by half a period. $\theta_{Mi}$, $\theta_{Ni}$ and $F_{Ni}$ are indicated by the red solid line, the blue dashed line and the purple dashed line, respectively. The displacement curve of the robot’s center of mass on the *X*, *Y* and *Z* axes is shown in Figure 5. The displacements in the *X*, *Y* and *Z* directions are indicated by the red solid line, the blue dashed line and the purple dashed line, respectively. It can be seen from the Figure that the displacement in the *Z* direction was the largest, because the robot moved in a vertical direction. The displacement in the *Y* direction was zero, because there was no motion in the *Y* direction, and the displacement in the *X* direction was almost zero, because there was also no motion in the *X* direction. The very small displacement change in the *X* direction was due to a motion error caused by slipping and other factors. The diagonal gait adopted in straight walking is shown in Figure 6. It can be seen from the Figure that a better movement effect was achieved by the planned gait.

### 3.5. Turning Motion

In straight walking, the angles of the diagonal legs are equal, while, in the turning process, the final turn occurs through the speed difference between the diagonal legs. There is a big difference between them, and control over the turning motion is complicated. There were two main differences between the robot’s turning gait and its straight-line gait. On the one hand, the swing angle of the robot’s diagonal telescopic leg joint is not consistent when turning. On the other hand, the turning radius of the robot’s quadruped is not equal, so the distance between the robot’s turning trajectory and the planned center of the circle is always the same when planning the trajectory of the telescopic foot joint.

When the robot moves according to the turning gait, $K_{0}=0$ and $K_{S}=0$, and Equation (3) can be simplified as (5). By adjusting $A_{D}$ independently in Equation (5), the robot was able to turn and walk.

**Figure 7:**
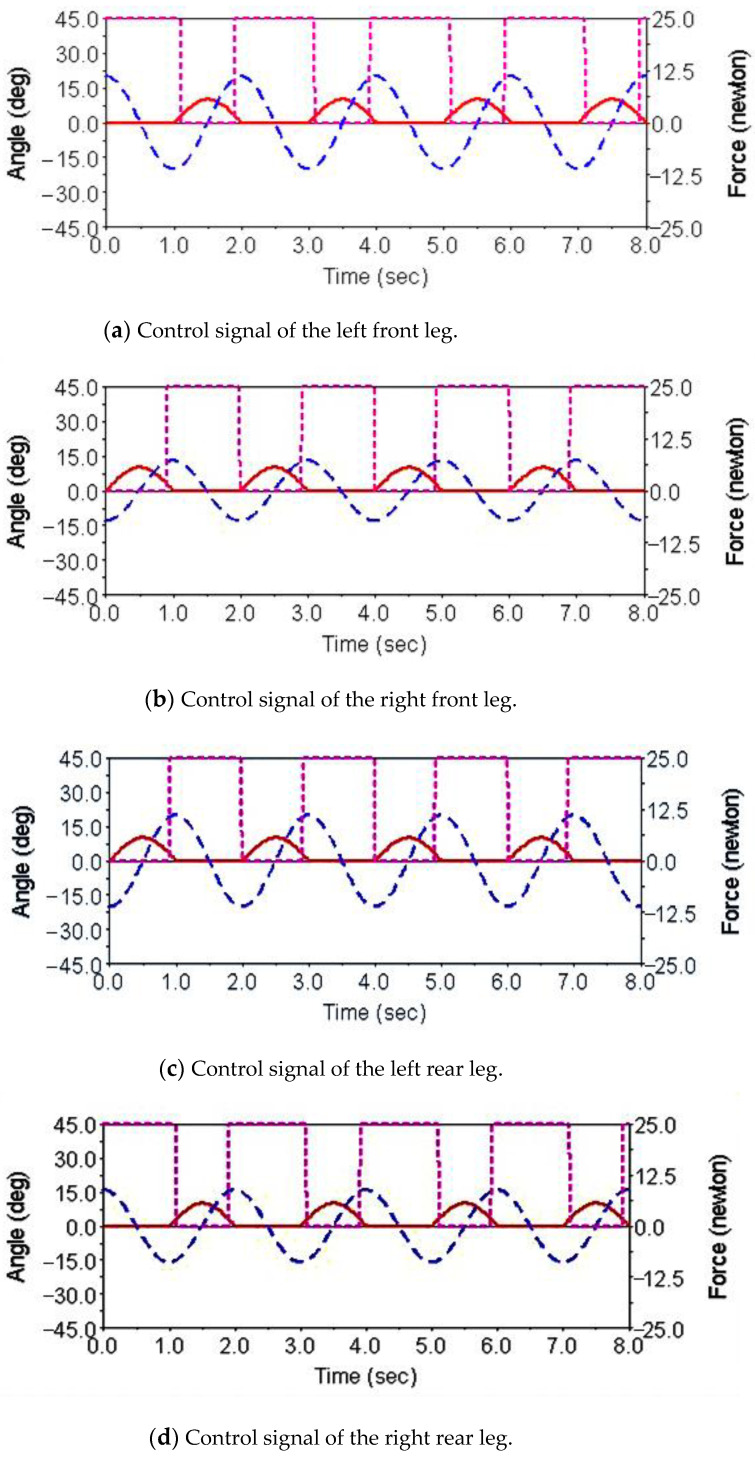
Diagonal gait for turning motion in ADAMS.

**Figure 8:**
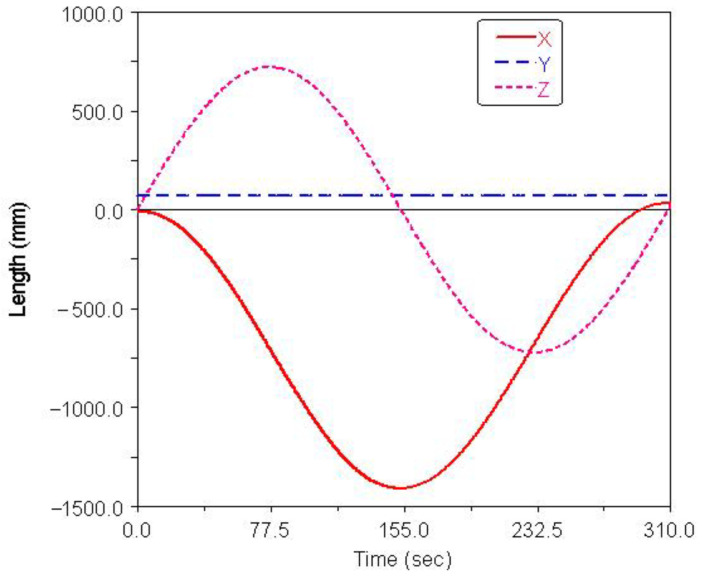
Centroid displacement curve of turning gait.

**Figure 9:**
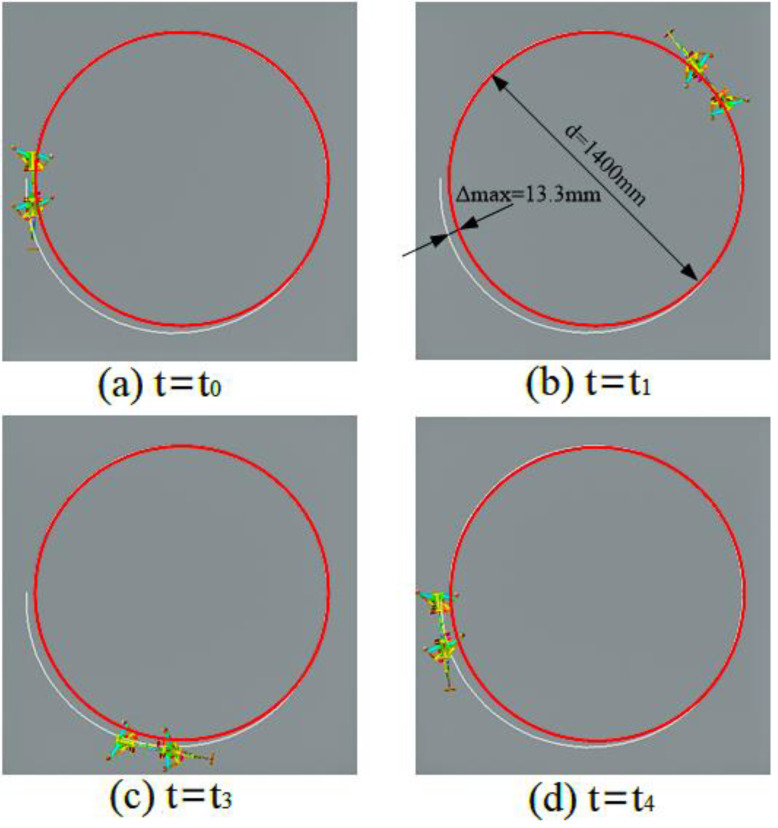
Turning movement of the robot using a diagonal gait in ADAMS in different moments.

Gait simulation was also carried out in the automatic dynamic analysis of mechanical systems (ADAMS) environment. The control signals for each foot are shown in Figure 7, among them, $A_{D1}=A_{3}/A_{1}=0.8$, $A_{D2}=A_{4}/A_{2}=0.65$. It can be seen from the Figure that the robot moved according to the corresponding gait. The displacement curve of the center of mass of the robot on the *X*, *Y* and *Z* axes is shown in Figure 8. It can be seen from the Figure that the displacement in the *X* and *Z* directions changed with time, because the robot moved in the *X* and *Z* planes. The displacement in the *Y* direction was zero, because there was no motion in the *Y* direction. The effect picture of the robot walking with a turning gait is shown in Figure 9. It can be seen from the Figure that a better movement effect was achieved according to the planned gait. Due to friction and other factors, there was a relative slip between the electromagnetic sucker and the wall, and the maximum deviation between the trajectory of the mass center and the theoretical planning trajectory, that is, a circle with a diameter of 1400 mm is 13.3 mm.

### 3.6. Striding over Obstacles

When the robot walked on the plane at a normal rhythm, the oscillation amplitude of the leg-lifting joint was small, which was beneficial for reducing energy consumption and increasing the adsorption stability of the robot. When obstacles are detected in front of the robot, the obstacle gait response switch ($K_{0}=1$) can be triggered, and Equation (3) can be simplified as follows:

(6) $$\{\begin{array}{c} \theta_{Mi}=\{\begin{array}{c} A_{M}\sin(\frac{2\pi t}{T}+\varphi_{i})+K_{0}A_{0}\sin(\frac{2\pi (t-t_{0})}{T_{0}})\;\;\;\;\;\;\;\;\;\;\;K_{M}=0\\ \max(A_{M}\sin(\frac{2\pi t}{T}+\varphi_{i})+K_{0}A_{0}\sin(\frac{2\pi (t-t_{0})}{T_{0}}),0)\;\;\;\;\;\;\;\;K_{M}=1 \end{array}\\ \theta_{Ni}=A_{N}\sin(\frac{2\pi t}{T}+\varphi_{i}+\varphi_{N})\\ F_{i}=\{\begin{array}{c} F(\mathrm{AINT}((t+\frac{4\varphi_{S}}{T}\pm \frac{\Delta t}{2})\mathrm{mod}T))\;\;\;\;\;\;\;\;\;\;K_{H}=0\\ 0\;\;\;\;\;\;\;\;\;\;\;\;\;\;\;\;K_{H}=1 \end{array} \end{array}$$

**Figure 10:**
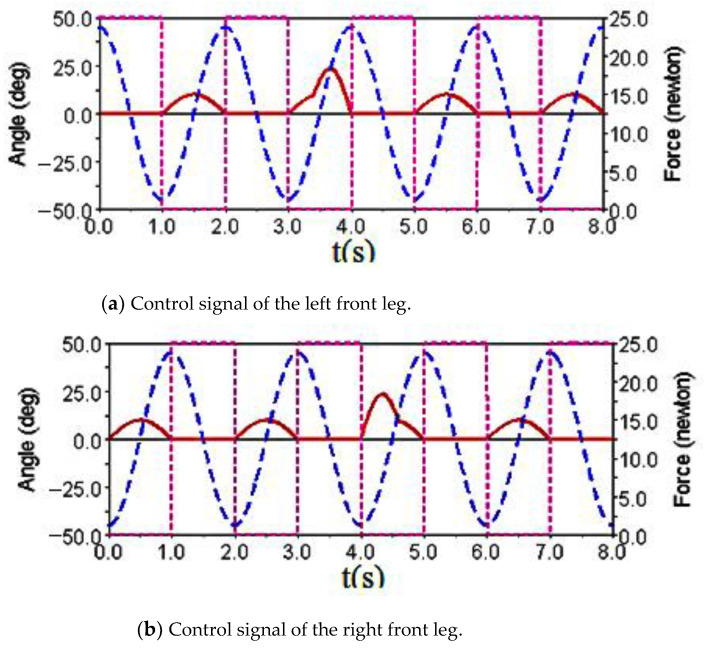

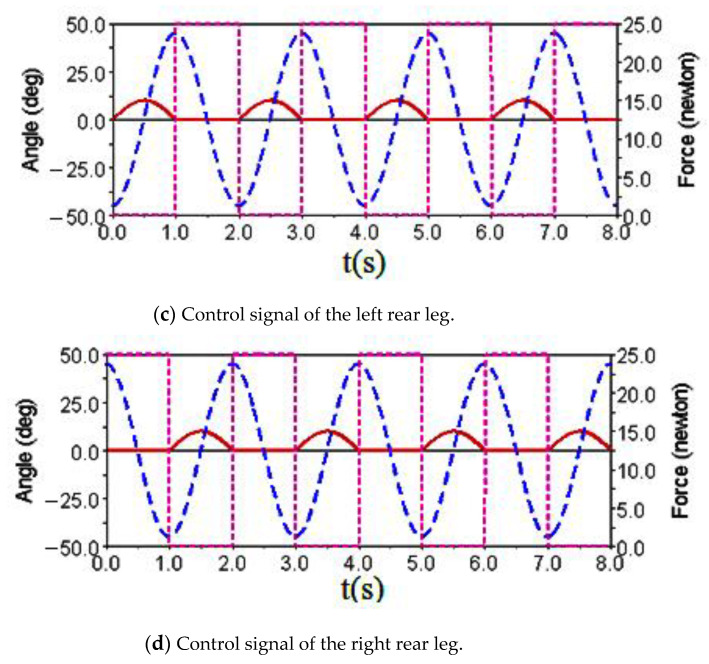
Robot leg control signals using the diagonal gait for crossing over obstacles.

**Figure 11:**
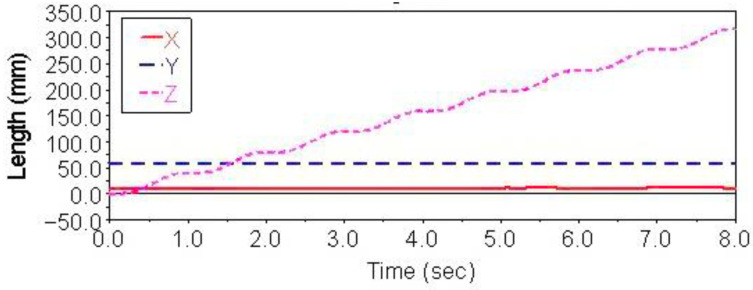
Centroid displacement curve for crossing over obstacles.

**Figure 12:**
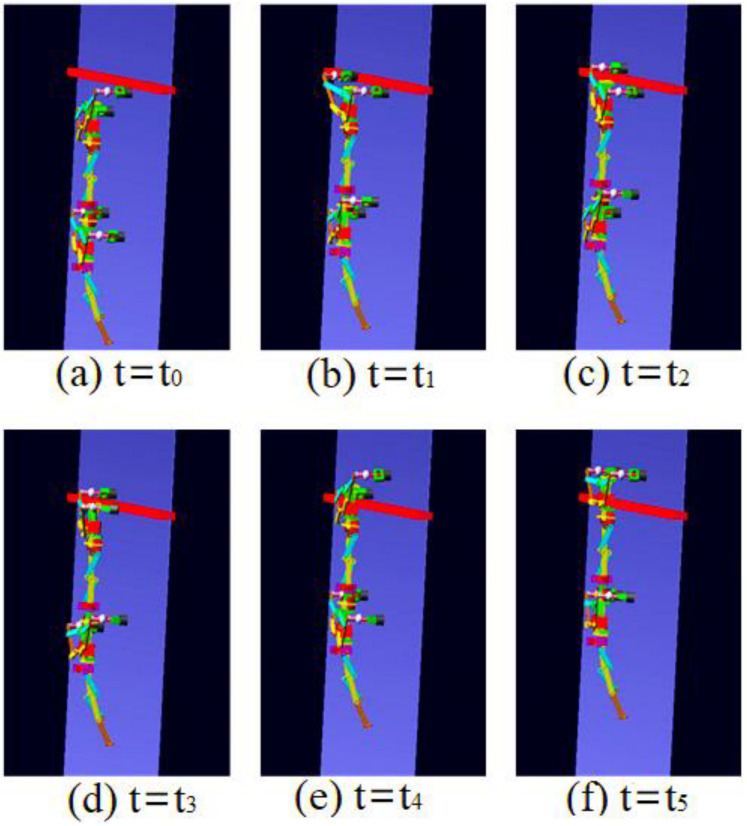
Movement of the robot using diagonal gait when crossing obstacle in ADAMS in different moments.

The control signal for the robot striding over the obstacle is shown in Figure 10. In the period of 0–3.4 s, the robot walked with a diagonal gait rhythm; the response switch ($K_{0}=1$) of the barrier-crossing gait was triggered by a high-level decision-making system when t = 3.4 s. The robot strode over the obstacle by increasing the swing of its left front leg, which then rapidly fell to the ground. At the same time, the right front leg and other legs strode over obstacles in the same way. After crossing the obstacles, the robot replaced the barrier-crossing gait and walked with a diagonal gait. The displacement curve of the center of mass of the robot on the X, Y and Z axes is shown in Figure 11. It can be seen from the Figure that the displacement in the Z direction was the largest, because the robot moved in a vertical direction, and the displacement in the X direction was zero, because there was no motion in the X direction. The very small displacement change in the Y direction was due to a motion error caused by slipping and other factors. The effect picture of the robot, t, striding over obstacles is shown in Figure 12. It can be seen from the Figure that a better movement effect was achieved according to the planned gait.

### 3.7. Climbing Step

The climbing step is based on linear motion. The robot adapted to the step by adjusting the front and rear foot joint to increase or decrease the rhythmic oscillation offset. The foot on the step took the positive offset, while the foot under the step stayed the same or took the negative offset. Equation (3) can be simplified as follows:

(7) $$\{\begin{array}{c} \theta_{Mi}=\{\begin{array}{c} A_{M}\sin(\frac{2\pi t}{T}+\varphi_{i})+K_{S}(A_{S}+K_{S}^{'}A_{S}\sin(\frac{2\pi (t-t_{S})}{T}))\;\;\;\;\;\;\;\;\;\;K_{M}=0\\ \max(A_{M}\sin(\frac{2\pi t}{T}+\varphi_{i})+K_{S}(A_{S}+K_{S}^{'}A_{S}\sin(\frac{2\pi (t-t_{S})}{T})),0)\;\;\;\;\;\;\;\;\;K_{M}=1 \end{array}\\ \theta_{Ni}=A_{N}\sin(\frac{2\pi t}{T}+\varphi_{i}+\varphi_{N})\\ F_{i}=\{\begin{array}{c} F(\mathrm{AINT}((t+\frac{4\varphi_{S}}{T}\pm \frac{\Delta t}{2})\mathrm{mod}T))\;\;\;\;\;\;\;\;\;\;\;K_{H}=0\\ 0\;\;\;\;\;\;\;\;\;\;\;\;\;\;\;\;K_{H}=1 \end{array} \end{array}$$

**Figure 13:**
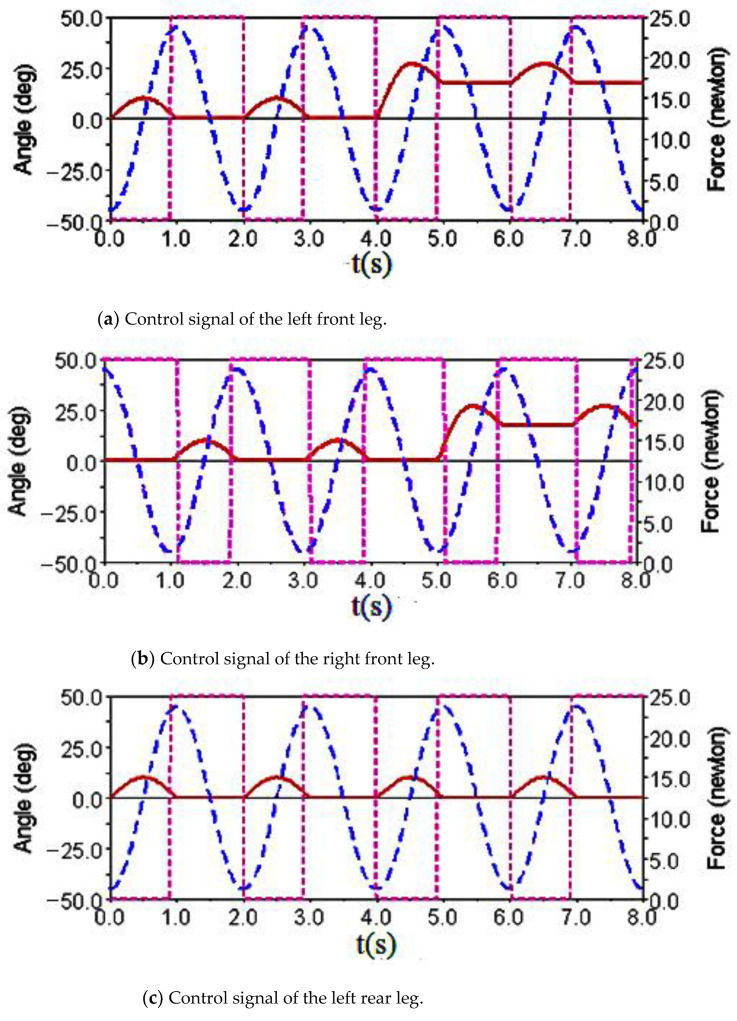

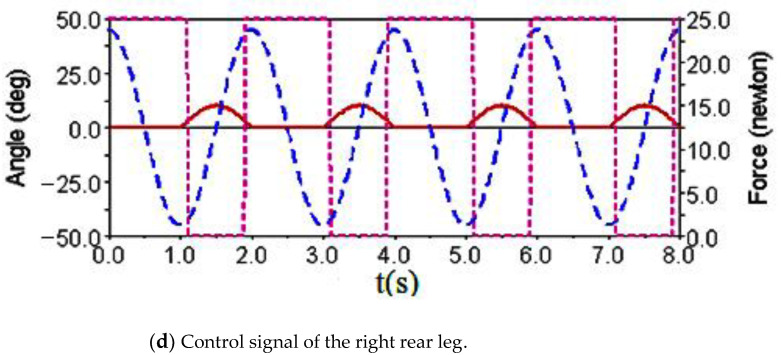
Robot leg control signals for the diagonal gait with the climbing step.

**Figure 14:**
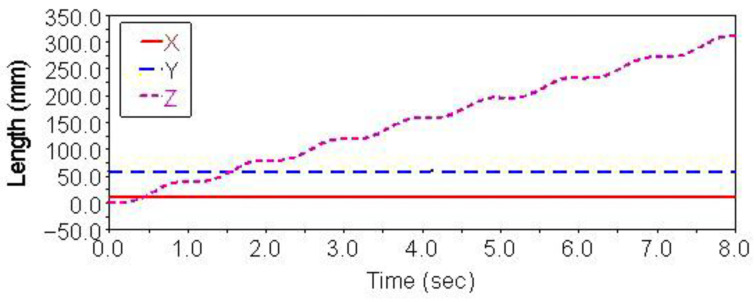
Centroid displacement curve of climbing step.

**Figure 15:**
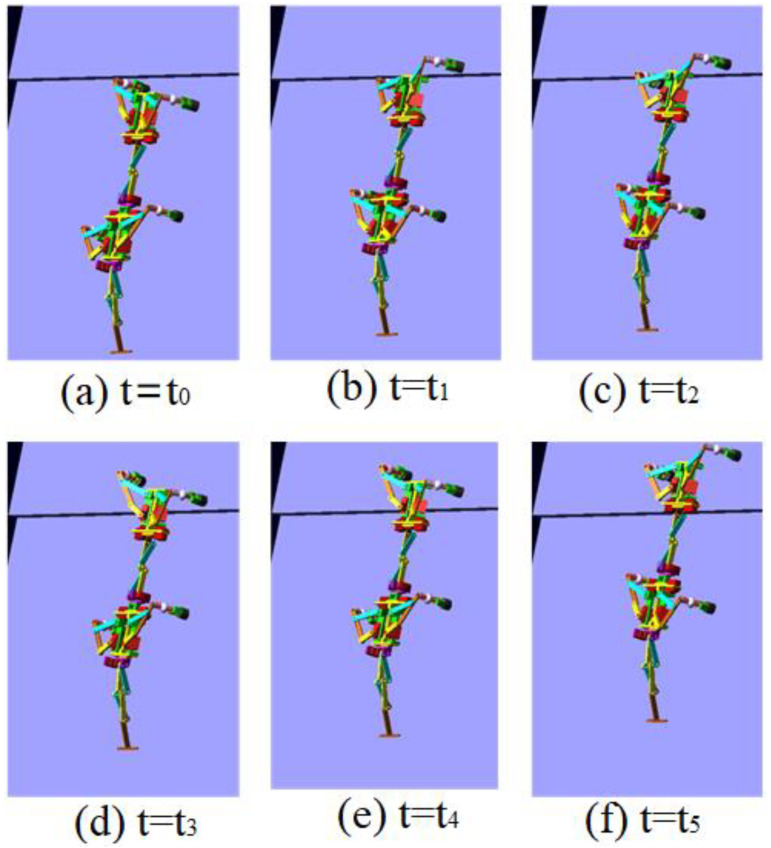
Movement of the robot using diagonal gait when climbing step in ADAMS in different moments.

The control signals for each foot when the robot climbed the step surface are given in Figure 13. In the period of 0–4 s, the robot walked with a diagonal gait rhythm; the response switch ($K_{S}$) of the climbing step gait was triggered by a high-level decision-making system when t = 4 s. The robot strode up the step and maintained the offset value corresponding to the step height by increasing the swing ($A_{S}$) of its right front leg. At the same time, the left front leg of the robot strode up the step in the same way; after stepping up the step, the robot stopped adopting the step gait by crossing the obstacle and walked with the original diagonal gait until the back foot strode up the step and restored the offset value to zero. The displacement curve of the center of mass of the robot on the X, Y and Z axes is shown in Figure 14. It can be seen from the Figure that the displacement in the Z direction was the largest, because the robot moved in a vertical direction, and the displacement in the X direction was zero, because there was no motion in the X direction. The very small displacement change in the Y direction was due to a motion error caused by slipping and other factors. The effect picture of the robot, t, striding over obstacles is shown in Figure 15. It can be seen from the Figure that the robot successfully climbed the step according to the planned gait.

## 4. Experimental Study

### 4.1. Experimental Conditions

**Figure 16:**
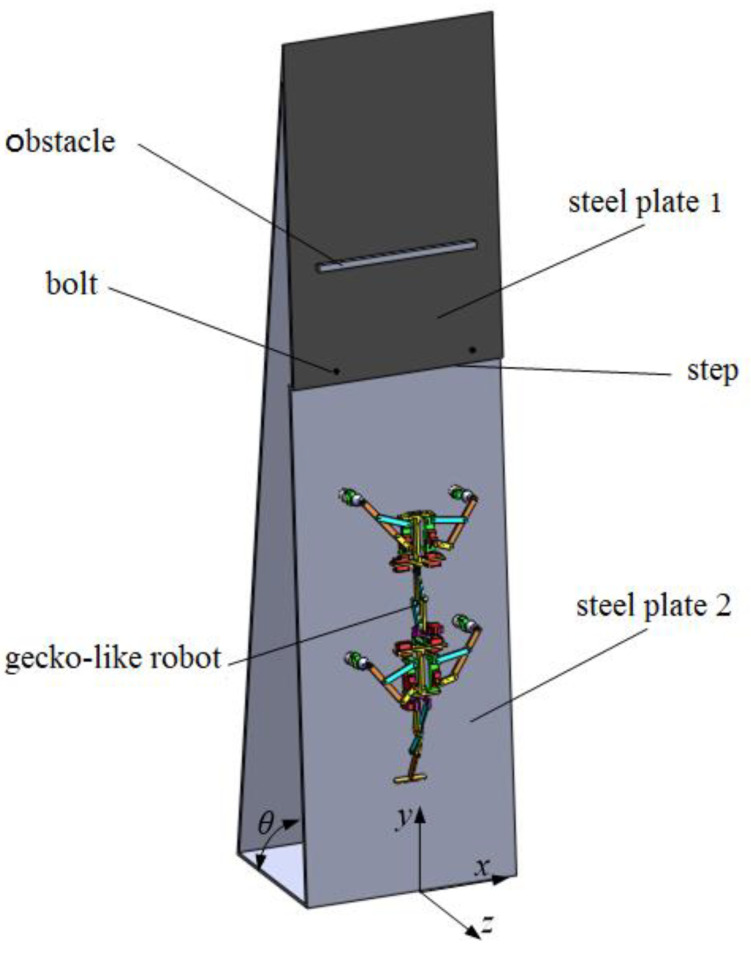
Experimental environment.

The experimental system, shown in Figure 16, included steel plate 1, steel plate 2, an obstacle and the robot. Steel plate 1 and steel plate 2 were made of ordinary carbon steel with a width of 400 mm, and the inclination (θ) between the steel plate and the horizontal plane could be adjusted. Steel plate 2 and steel plate 1 were connected by bolts to form a step with a height of 8 mm. The size of the obstacle was 12 mm × 12 mm × 220 mm. The adsorption method was electromagnet adsorption, and the maximum adsorption force of the electromagnet was 25 N.

### 4.2. Experimental Results

**Figure 17:**
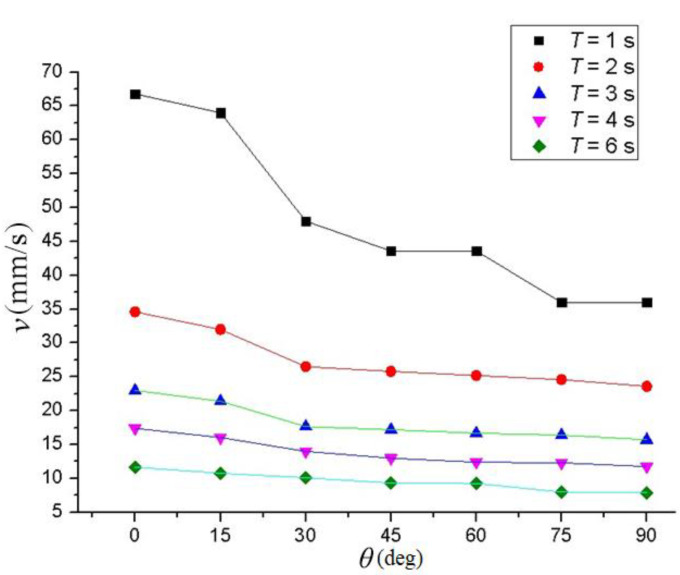
The influence of the oscillation period and slope gradient on the walking speed of the robot.

Figure 17 shows the experimental results for the walking speed of the robot under the different CPG parameters. The speed curve was obtained by quadratic interpolation. The discontinuous growth of the speed value was mainly caused by the slip of the robot’s electromagnetic foot and the consequent measurement error, in which T is the oscillation period and θ is the slope gradient. It can be seen from the Figure that the time period was fixed, the walking speed of the robot decreased along with the increase in slope gradient, and the longer the oscillation period, the faster the robot walked.

**Figure 18:**
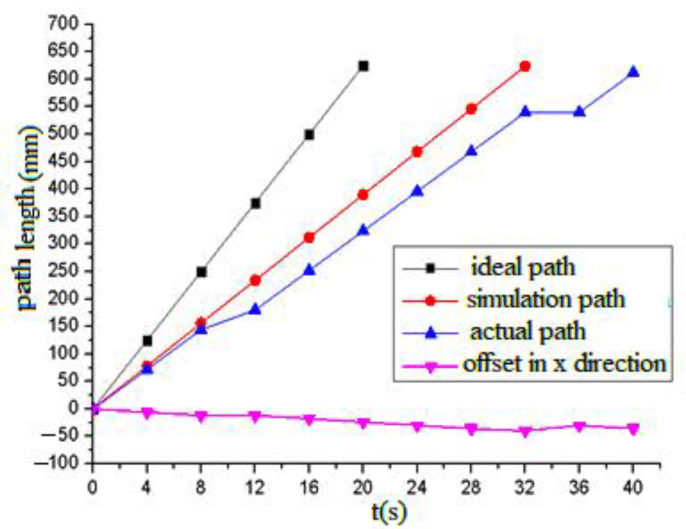
Comparison of the ideal path, the planned path and the actual path.

Figure 18 shows the comparison curve of the ideal path, the simulation path and experimental path without slips when t equals two seconds and θ equals 90°. The ideal path, simulation path, experimental path and offset in the x direction are indicated by the black solid line with squares, the red line with dots, the blue line with arrows and the pink line with downward arrows, respectively. It can be seen from the Figure that the ideal path and simulation path were linear with time, and there was a certain deviation between them. Due to the influence of the slip and other factors, the deviation between the experimental path and the simulation path was larger, especially for crossing obstacles, where the deviation changed greatly, but this did not affect the actual experimental effect. In 12 s and 36 s, the robot crossed the steps or obstacles by increasing the swing of the leg-lifting joint. At this time, the electromagnetic chuck and the steel plate formed a certain angle, resulting in a decrease in the magnetic induction intensity, a fluctuation in the adsorption force and the relatively large slip of the robot.

**Figure 19:**
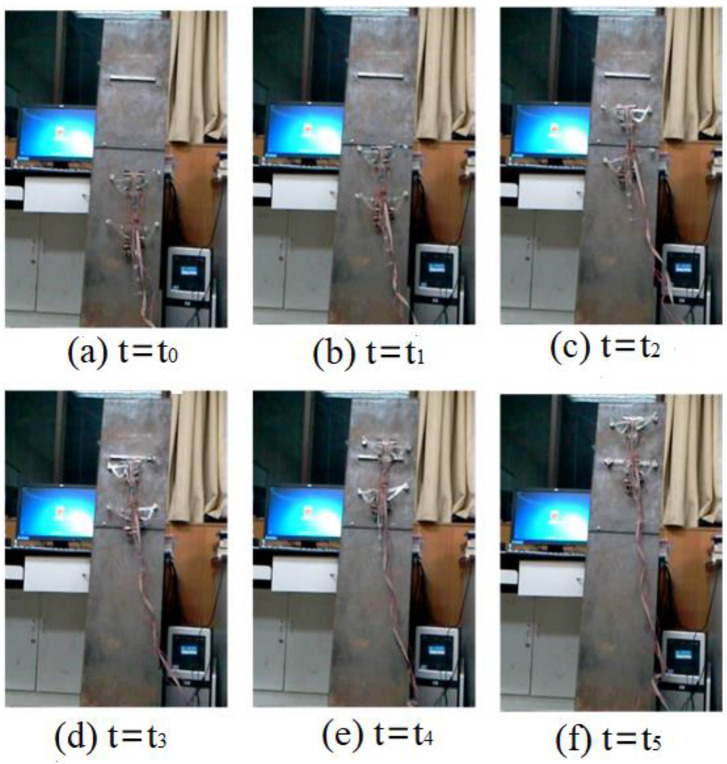
Video screenshot of the robot walking in a 90° complex environment in different moments.

Video screenshots of the gecko-like robot walking in plane, climbing steps and crossing obstacles are shown in Figure 19. In the actual experimental environment, by changing the experimental method and constantly optimizing various parameters, the experimental effect was improved. After many experiments, the gecko-like robot steadily walked, climbed steps and crossed obstacles in the vertical plane.

### 4.3. Experimental Analysis

According to the experimental results, the forward speed of the gecko-like robot decreased with the increasing oscillation period and decreased as slopes increased. The robot demonstrated a better climbing effect and environmental adaptability when the oscillation period was 1–6 s. When the slope was 90°, the maximum climbing speed was 38 mm/s. In the experiment, the adsorption time of the electromagnet was controlled by the current on/off method. Due to the remanence and slip of the electromagnet, the adsorption and release of the electromagnet lagged behind the on–off time of the current, so the oscillation period *T* could not be too short. Additionally, the walking line of the robot slightly deviated from the predetermined track due to the manufacturing accuracy, slip and deviation of gravity of the robot, and the largest offset was 31 mm. If the robot structure is further optimized and flexible bionic feet are installed in a later stage, the adsorption effect can be improved, the slip can be reduced, and the robot will have better adaptability to complex environments.

## 5. Conclusions

In this study, a motion control network model based on a central neural pattern generator was designed for the motion control of a gecko-like robot in a complex environment. The gecko-like robot achieved a stable walking rhythm in a diagonal gait using CPG. Furthermore, we designed the CPG network model to help the robot cross obstacles and climb steps. The feasibility and validity of the CPG motion control model were verified by ADAMS in simulations and experiments. Because the coupling relationship and the output signal of the CPG network can be adjusted quickly and stably, this method is universally applicable to the motion control of common quadruped robots and multilegged robots.

The simulation environment we used, which is not included with the perceptual system and advanced decision system, is relatively simple. Therefore, the switching time between gait triggers is preset through the program. To substantially increase the robot’s adaptability to the environment and to achieve steady climbing and walking in complex terrains and in dynamic environments, future work will include developing obfuscation and senior decision-making. It is also our aim to incorporate the robot control and multi-target tracking tasks in future research [33,34].

## Figures and Tables

**Table 1:** Main parameters of the gecko-like robot.

Size	470 mm × 208 mm × 470 mm
Quality	421.5 g (not including the control part)
Swing range of lifting joint	±25°
Extension and contraction range of leg joint	±45°
Taper of leg driven joint	15°
Swing range of lumbar joint	±90°
Swing range of tail joint	±90°

**Table 2:** Definitions and values of the parameters of the CPG network.

Symbol	Meaning	Value of Simulation/Experiment
$\theta_{Mi}$	joint angle of lifting leg	
$\theta_{Ni}$	joint angle of extension leg joint angle	
$F_{i}$	adsorption capacity of foot	
$A_{M}$	oscillation amplitude of lifting leg	10°
$A_{N}$	oscillating amplitude of telescopic leg	45°
$\theta_{i}$	delayed phase of each foot	π
$\theta_{N}$	phase difference of the telescopic leg joint relative to the lifting leg joint	π2
$\theta_{s}$	phase difference between sucker and retractable joint	π/2
T	oscillation period	2 s
$T_{o}$	oscillation period of obstacle reflection	1.6 s
$t_{0}$	obstacle reflex generation moment	3.4 s
$t_{s}$	moment of reflection generation of step surface walking	3.4 s
Δt	simultaneous adsorption time of feet	0.1 s
F	adsorbing force of suction cup	25 N
$A_{o}$	amplitude of reflection oscillation	15°
$A_{s}$	walking angle of stepped surface	15°
$A_{D}$	turning gait coefficient	0~1
$K_{o}$	response switch of barrier gait	0 or 1
$K_{s}$	response switch of step gait	0 or 1
$K_{s}^{,}$	oscillator switch of step gait	0 or 1
$K_{M}$	half/full wave selection switch	0 or 1
$K_{N}$	adsorption selection switch	0 or 1
AINT	rounding	
mod	mod	

**Table 3:** Values of contact forces between robot suction cup and supporting surface.

Definition	Values
Stiffness	1.0 × 10^5^ N/mm
Force Exponent	2.2
Damping	10 Ns/mm
Penetration Depth	0.1 mm
Static Friction Coefficient	0.7
Static Friction Slip Velocity	0.3 mm/s
Dynamic Friction Coefficient	0.1
Dynamic Friction Transition Velocity	1 mm/s

## Data Availability

Not applicable.
